# A SNP transferability survey within the genus *Vitis*

**DOI:** 10.1186/1471-2229-8-128

**Published:** 2008-12-16

**Authors:** Silvia Vezzulli, Diego Micheletti, Summaira Riaz, Massimo Pindo, Roberto Viola, Patrice This, M Andrew Walker, Michela Troggio, Riccardo Velasco

**Affiliations:** 1 IASMA Research Centre, Via E. Mach 1, 38010 San Michele all'Adige (TN), Italy; 2 Department of Viticulture and Enology, University of California, One Shields Avenue, CA-95616 Davis, USA; 3 UMR 1097, DIA-PC Equipe Génétique Vigne, INRA-Supagro, 2 place Viala, F-34060 Montpellier, France

## Abstract

**Background:**

Efforts to sequence the genomes of different organisms continue to increase. The DNA sequence is usually decoded for one individual and its application is for the whole species. The recent sequencing of the highly heterozygous *Vitis vinifera *L. cultivar Pinot Noir (clone ENTAV 115) genome gave rise to several thousand polymorphisms and offers a good model to study the transferability of its degree of polymorphism to other individuals of the same species and within the genus.

**Results:**

This study was performed by genotyping 137 SNPs through the SNPlex™ Genotyping System (Applied Biosystems Inc.) and by comparing the SNPlex sequencing results across 35 (of the 137) regions from 69 grape accessions. A heterozygous state transferability of 31.5% across the unrelated cultivars of *V. vinifera*, of 18.8% across the wild forms of *V. vinifera*, of 2.3% among non-*vinifera Vitis *species, and of 0% with *Muscadinia rotundifolia *was found. In addition, mean allele frequencies were used to evaluate SNP informativeness and develop useful subsets of markers.

**Conclusion:**

Using SNPlex application and corroboration from the sequencing analysis, the informativeness of SNP markers from the heterozygous grape cultivar Pinot Noir was validated in *V. vinifera *(including cultivars and wild forms), but had a limited application for non-*vinifera Vitis *species where a resequencing strategy may be preferred, knowing that homology at priming sites is sufficient. This work will allow future applications such as mapping and diversity studies, accession identification and genomic-research assisted breeding within *V. vinifera*.

## Background

The number of genomes sequenced continues to increase. These sequences are usually decoded for one individual, but their application is considered for the entire species and even within the genus. Sequencing projects have been completed for a number of herbaceous plants, namely thale cress (*Arabidopsis thaliana*, [1]) and rice (*Oryza sativa*, [2]), while for woody species, such as black cottonwood poplar (*Populus trichocarpa*, [3]) and papaya (*Carica papaya*, [4]), a draft assembly is available. The high commercial value of grape (*Vitis vinifera *L.) gave rise to the funding of two sequencing programs on a near-homozygous line [5] and a highly heterozygous cultivar [6]. The genome sequencing of this latter genotype resulted in several thousand polymorphisms and therefore provided a good model to study the transferability of its polymorphism content across individuals within *V. vinifera *and within *Vitis*.

Grape is one of the oldest and most important perennial crops in the world. Its cultivation is concentrated in regions with a Mediterranean-type climate but it is grown in most temperate regions. The vast majority of the world's grapes are cultivars of *V. vinifera *subsp. *sativa *(from here reported as *V. vinifera *cultivars), which are used as a source of fresh fruit and raisins, and fermented to make wine and distilled beverages. Cultivated grapes were domesticated from the wild *V. vinifera *subsp. *sylvestris *[7], which was once distributed widely from the Middle East to Western Europe [8]. This range was greatly restricted by the introduction of the mildew diseases and grape phylloxera from North America in the mid-1800s, to which *V. vinifera *subsp. *sylvestris *is highly susceptible [9]. The genus *Vitis *is unique among the 15 (GRIN database;  recognized in the family *Vitaceae *in having 38 chromosomes that form 19 bivalents at meiosis. Most other related genera in *Vitaceae*, including *Muscadinia*, 2n = 40, have multiples of 10 chromosomes. Often classified as a subgenus of *Vitis*, *Muscadinia *has three species native to the southern USA and eastern Mexico, *M. rotundifolia *is the only one cultivated. The genus *Vitis *consists of about 60 inter-fertile primarily Northern Hemisphere species with about 30 in America and 30 in Asia. Because of their resistance to a wide range of pests and pathogens, several *Vitis *species have been extensively used for breeding rootstocks and inter-specific hybrids. *Muscadinia rotundifolia *has been hybridized with *V. vinifera *in efforts to combine the exceptional disease and pest resistance of *M. rotundifolia *with the high fruit quality of *V. vinifera *[9,10].

Grape breeding is a relatively slow process, which can take up to 25 years or more to produce a new cultivar, and perhaps longer when combining desirable fruit and disease resistance traits. This breeding for pest and disease resistance while combining high fruit quality is critical for long-term health of the viticulture industry and the reduction of the current intensive and wide-spread use of pesticides. The use of genetic markers that are tightly linked to horticultural traits has allowed breeders to accelerate the breeding process through marker-assisted selection (MAS) in plant species [e.g. [11]]. The first examples of this process in grape utilized RAPD and AFLP markers, which were converted into more useful SCAR markers [e.g. [12-15]]. More recently, SSR or microsatellite markers have been successfully employed in marker-assisted breeding for table grapes [16], wine grapes and rootstocks [17]. Because of their multiallelic and reproducible nature, SSRs have been extensively used in mapping studies [e.g. [18,19]] and for genome anchoring [5,6]. To date more than 500 grape SSRs are publicly available and are described in the NCBI databases dbSTS and UniSTS .

SNPs, dichotomous (biallelic) markers, have been developed in many species [e.g. [20,21]] including grape where they were derived from BAC and EST libraries and used successfully to build genetic maps [22,23] and to anchor them to a physical map [24]. In addition, SNPs identified in grape gene sequences have been employed in genetic diversity studies [25,26] and *linkage disequilibrium *analyses [27,28]. Moreover, the recent decoding of the grape genome sequence in the heterozygous Pinot Noir cultivar provided the grape research community with 1,700,000 SNPs from coding and non-coding regions [6]. Different strategies have been applied in grape for SNP detection and genotyping, including low to mid [29] and high throughput [30] methods. However, most of the SNP discovery and application has been limited to *V. vinifera*. The use and transferability of SNPs across *V. vinifera *has been restricted to a few cultivars [23] and to a few wild forms [27]. In addition to the development of markers for MAS, knowledge on transferability of SNPs is required to allow the identification of useful alleles for diversity and association studies. To date, a comprehensive study of SNP use and transferability across species within *Vitis *has never been attempted.

The primary goal of this study was to assess the use and transferability of SNPs discovered from the heterozygous Pinot Noir [6] within the species *V. vinifera *and within the genera *Vitis *and *Muscadinia *to validate the use of Pinot Noir genome-based SNP markers for future grape improvement programs using diverse genetic resources. This study was performed by genotyping 137 SNPs through the SNPlex™ Genotyping System (Applied Biosystems Inc.) and by confirming the SNPlex results sequencing 35 (of the 137) regions on 69 accessions.

## Methods

### Plant material and genomic DNA extraction

Genomic DNA (gDNA) of 71 genotypes was isolated from 50 to 100 mg of young leaves. After freeze-drying, leaf material was ground using the MM 300 Mixer Mill (Retsch Inc., Haan, Germany) and DNA extraction was performed using the DNeasy 96 Plant Mini Kit (Qiagen, Valencia, California, USA) according to the manufacturer's protocol.

The set of 71 genotypes consisted of: i) 24 *V. vinifera *cultivars housed at IASMA (Italy), including the sequenced Pinot Noir clone ENTAV 115; this set included 7 varieties related to Pinot Noir at the 1^st ^degree level (Pinot Noir is a parent), 4 varieties related to Pinot Noir at the 2^nd ^degree level (Pinot Noir is a grand-parent) and 12 varieties unrelated to Pinot Noir; ii) 10 accessions of *V. vinifera *subsp. *sylvestris *housed at Vassal INRA-Montpellier (France); and iii) 37 non-*vinifera *accessions housed at UC Davis (California), representing 25 *Vitis *species and 2 accessions of *M. rotundifolia *[see Additional file 1].

### Whole Genome Amplification

Ten ng of gDNA were amplified by whole genome amplification (WGA) [31] using the GenomiPhi V2 DNA Amplification Kit (GE Healthcare, Little Chalfont, Buckinghamshire, United Kingdom) according to the manufacturer's protocol. The success of the WGA reaction and the absence of product in the negative control samples were assessed by agarose gel electrophoresis.

### SNPlex assay and data analysis

The SNPlex (Applied Biosystems Inc., [32]) assay was carried out on 1 μl (from 45 to 225 ng) of fragmented GenomiPhi amplified gDNA (WGA-DNA) diluted to a final volume of 12 μl and air dried in the dark.

Six SNP sets, for a total of 225 validated electronic SNPs (eSNPs), were chosen based on the number of validated SNPs over the total eSNPs in the Pinot Noir clone ENTAV 115 [30] (dbSNP NCBI Build 128). The SNPlex analysis was carried out according to the manufacturer's protocol modified for the amount of PCR product used in the hybridization cycles (3 μl instead of 1.5 μl). The samples were run on a 3730 × l DNA Analyzer (Applied Biosystems Inc.) and the data were analyzed using the Gene Mapper v.4.0 software (Applied Biosystems Inc.). Genotype analysis was performed based on the SNPlex_Rules_3730 method following factory default settings.

### Sequencing analysis

Ca. 450 bp (short-range) of flanking region for each of the 40 target SNPs [see Additional file 2] were sequenced in all 71 genotypes in order to confirm the SNPlex results and to discover all possible polymorphic sites (SNPs and In/dels). The selected 40 SNPs were scattered across the 19 grapevine chromosomes. Twenty of them were present in coding (inside predicted genes: exons and introns) regions and 20 corresponded to non-coding (outside predicted genes) regions ( – IASMA Genome Browser). In addition, ca. 850 bp (long-range) encompassing each of three target SNPs (SNP6038, SNP6082, and SNP6132) were sequenced in all 71 genotypes in order to investigate the short-range primer regions and to find additional mutations.

For both short- and long-sequencings, PCR primers were designed based on the Pinot Noir genomic sequence [6], using the Primer3 software [33] according to the following criteria: i) forward and reverse primers 200–250 bp or 500 bp upstream and downstream in respect to the target SNP; ii) primer size between 18 and 25 bases; iii) primer melting temperature (Tm) between 59 and 61°C; iv) alignment score and global alignment score for self-complementarity and complementarity between primer pairs ranging from 8 to 13 [see Additional file 2].

Subsequently, the 40 genomic regions were amplified in all 71 genotypes. PCR reactions were assembled using the following conditions: 1–20 ng of gDNA, 1× PCR buffer (Qiagen, Valencia, California, USA), 0.2 mM each dNTP, 0.4 μM of each primer, 1 U HotStarTaq DNA polymerase (Qiagen, Valencia, California, USA), and water to a final volume of 12.5 μl. The DNA amplifications were performed using a 15 min initial denaturation/activation step, followed by 30 cycles at 94°C for 30 sec, 57°C for 30 sec, and 72°C for 1 min, with a final extension step of 10 min at 72°C. PCR products were assessed by electrophoresis in 1.5% agarose gel and visualized by ethidium bromide staining. In order to remove unincorporated dNTPs and primers during the amplification reaction, the positive amplicons were purified through MultiScreen384PCR Cleanup plate (Millipore, Carrigtwohill, Co. Cork, Ireland).

The sequencing of the PCR products was carried out using the BigDye Terminator Cycle Sequencing Ready Reaction Kit v3.1 (Applied Biosystems Inc.) as follows: 2 μl of PCR purified products, 1X Sequencing buffer, 0.32 μM of forward primer, 1 μl of BigDye Terminator and deionized water to a final volume of 10 μl. The sequencing reactions were performed using a 2 min initial denaturation step, followed by 25 cycles at 96°C for 10 sec, 50°C for 5 sec and 60°C for 4 min and then purified from unincorporated primer and BigDye excess through Multiscreen384SEQ Sequencing reaction Cleanup Plate (Millipore, Carrigtwohill, Co. Cork, Ireland). Capillary electrophoresis of the purified products was performed on a 3730 × l DNA Analyzer (Applied Biosystems Inc.). DNA sequence electropherograms were aligned with the Pregap4/Gap4 software package (Staden Package, [34]) and used to scan all polymorphic sites in the 71 genotypes.

## Results

### SNPlex assay and data analysis

Out of the 225 analysed eSNPs on 71 genotypes, 137 satisfied the quality value (a mean of 98% genotype call rate per SNPset) and were therefore considered successful, while 88 regions were unsuccessful. The technical transferability was thus of 61% (137 SNPs) and corresponded to SNPs with homologous flanking sequences.

The transferability in the strict sense, namely those SNPs that maintained the heterozygous state, was analysed based on the four grapevine sub-groups (24 *V. vinifera *cultivars, 10 *V. vinifera *subsp. *sylvestris *accessions, 35 non-*vinifera Vitis *accessions, and 2 *M. rotundifolia *accessions). This transferability was 41.9% in *V. vinifera *cultivars(ranging from 52.9% – among genotypes related to Pinot Noir at 1^st ^degree level to 31.5% among genotypes unrelated to Pinot Noir), 18.8% in *V. vinifera *wild forms, only 2.3% in non-*vinifera Vitis *species, and 0% in *M. rotundifolia *(Table 1).

**Table 1:** Transferability of heterozygosity (maintenance of the heterozygous state) in the four grapevine groups, totaling for 71 accessions.

**Genotypes**		Transferability of heterozigosity (%) *	
		**Mean**	**Min-Max**

***Vitis vinifera *sbs. *sativa***	average	41.9	0.8 – 70.0

	1st degree of parentage with PN	52.9	0.0 – 100.0

	2nd degree of parentage with PN	41.3	0.0 – 100.0

	unrelated with PN	31.5	0.0 – 75.0

			

***Vitis vinifera *sbs. *sylvestris***	average	18.8	0.0 – 60.0

			

**non-*vinifera Vitis *species**	average	2.3	0.0 – 34.0

			

***Muscadinia rotundifolia***	average	0.0	0.0

* number of heterozygous sites in each genotype group/total number of analyzed sites in each genotype group

**Figure 1:**
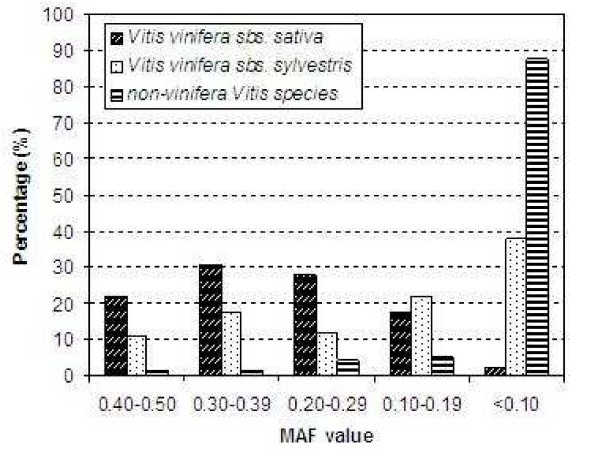
**MAF (Minor Allele Frequency) distribution of the 137 SNPs in three *Vitis *groups**.

For each SNP locus, the Minor Allele Frequency (MAF) value was also evaluated at the three *Vitis *levels (24 *V. vinifera *cultivars, 10 *V. vinifera *subsp. *sylvestris *accessions and 35 non-*vinifera Vitis *accessions). The analysis of MAF values showed that only 2.2% of the 137 SNPs displayed MAFs < 0.10 in *V. vinifera *cultivars, 38.0% in *V. vinifera *wild forms, and 87.6% in non-*vinifera Vitis *species, whereas 52.6% of 137 SNPs displayed MAFs ≥ 0.30 in *V. vinifera *cultivars, 28.5% in *V. vinifera *wild forms, and 2.9% in non-*vinifera Vitis *species (Figure 1). Three highly informative subsets of markers (those with MAF values ≥ 0.30), consisting of 72 SNPs for *V. vinifera *cultivars, 39 SNPs for *V. vinifera *wild forms, and 4 SNPs for non-*vinifera Vitis *species, were developed ("*" in Table S3). The first subset showed 50 *V. vinifera *cultivar specific SNPs, the second had 17 *V. vinifera *wild form specific SNPs, and the third revealed 1 non-*vinifera Vitis *species specific SNP ("x" in Table S3); 1 fully shared SNP marker (SNP0047) among the three subsets was identified [see Additional file 3].

### Sequencing analysis and mutation survey

In the sequencing analysis, amplicons corresponding to five SNPs (SNP0028, SNP0085, SNP6155, SNP7193, SNP8165) amplified in Pinot Noir but failed to amplify in 98% the studied genotypes. The remaining 35 successful regions – 20 corresponding to coding regions and 15 to non-coding regions – were sequenced in 69 *Vitis *genotypes. The two of *M. rotundifolia *accessions failed to amplify for all 35 regions. The identification of SNPs and In/del (both homozygous and heterozygous) was performed along the Staden Package alignments and resulted in the following conclusions:

i) 173 SNPs were discovered in the *V. vinifera *cultivars (106 in coding and 67 in non-coding regions) with an average of one SNP every 104 bp (one SNP every 117 bp in coding and every 91 bp in non-coding regions). Of these 173 SNPs, 62.4% (108) corresponded to transitions (A↔G, C↔T) and 37.6% (65) to transversions (A↔C, A↔T, C↔G, G↔T). In addition, 4 homozygous (2 in coding and 2 in non-coding regions) and 12 heterozygous In/dels (6 in coding and 6 in non-coding regions) were identified; when present, the average In/del frequency was of one every 360 bp.

ii) 116 SNPs were found in the *V. vinifera *wild forms (61 in coding and 55 in non-coding regions) with an average of one SNP every 129 bp (one SNP every 169 bp in coding and every 89 bp in non-coding regions). Of these 116 SNPs, 61.2% (71) corresponded to transitions and 38.8% (45) to transversions. In addition, 2 homozygous (1 in coding and 1 in non-coding regions) and 8 heterozygous In/dels (4 in coding and 4 in non-coding regions) were discovered; when present, the average In/del frequency was of one every 399 bp.

iii) 218 SNPs were identified in the non-*vinifera Vitis *species (124 in coding and 94 in non-coding regions) with an average of one SNP every 97 bp (one SNP every 112 bp in coding and every 82 bp in non-coding regions). Of these 218 SNPs, 70.6% (154) corresponded to transitions and 29.4% (64) to transversions. In addition, 17 homozygous (8 in coding and 9 in non-coding regions) and 38 heterozygous In/dels (22 in coding and 16 in non-coding regions) were identified; when present, the average In/del frequency was of one every 274 bp (Table 2).

**Table 2:** SNPs and In/dels identified in the 35 resequenced regions

**Genotypes**	**Coding regions**							
	**SNPs**						**In/dels**	

	**Transitions**		**Transversions**				**Heterozygous**	**Homozygous**

	A↔G	C↔T	A↔C	A↔T	C↔G	G↔T		

***Vitis vinifera *subs. *sativa***	37	29	12	15	6	7	6	2

***Vitis vinifera *subs. *sylvestris***	19	18	8	8	1	7	4	1

***non-vinifera Vitis *species**	41	43	13	15	9	3	22	8

	**Non-coding regions**							

	**SNPs**						**In/dels**	

	**Transitions**		**Transversions**				**Heterozygous**	**Homozygous**

	A↔G	C↔T	A↔C	A↔T	C↔G	G↔T		

***Vitis vinifera *subs. *sativa***	19	23	1	11	4	9	6	2

***Vitis vinifera *subs. *sylvestris***	14	20	0	8	6	7	4	1

***non-vinifera Vitis *species**	35	35	3	6	4	11	16	9

	**Total SNPs**			**Total In/dels**			**Total Mutations**	

***Vitis vinifera *subs. *sativa***	173			16			189	

***Vitis vinifera *subs. *sylvestris***	116			10			126	

***non-vinifera Vitis *species**	218			55			273	

Considering the three genotype groups, the size of the amplicons was generally conserved, except for a few non-*vinifera Vitis *species cases where short insertions (average 15 bp) were detected.

The observed heterozygosity (H_0_) was computed for each genotype based on all mutations (SNPs and In/dels) identified in the 35 resequenced loci. The *V. vinifera *cultivar Granoir (code VV19) was the most heterozygous (27%), while the accessions of *V. monticola, V. biformis *and *V. shuttleworthii *(code VS10, VS27, VS37) were the least (2%). The level of polymorphism could not be assessed in the two accessions of *M. rotundifolia *since the sequencing failed.

### Validation of the SNPlex genotyping system by sequencing

The sequencing analysis of the 35 SNP regions confirmed the genotype (target SNP) of Pinot Noir identified by SNPlex, which was in accord with Pindo *et al*. [30]. When the 2,415 sequencing data points (35 SNPs × 69 *Vitis *genotypes) were considered, 92 (3.8%) were inconsistent with the SNPlex results. Of these incongruous data 40 occurred in *V. vinifera *cultivars, 3 in *V. vinifera *wild forms, and 49 in non-*vinifera Vitis *species. Furthermore, 72 occurred in coding regions while 20 were present in non-coding regions. These data grouped in six classes: i) Class I: 52 (56.5%) were homozygous after sequencing, while SNPlex analysis indicated they were heterozygous; ii) Class II: 4 (4.3%) were heterozygous and one or more SNPs were identified close to the target SNP by sequencing, while SNPlex analysis indicated they were homozygous; iii) Class III: 17 (18.5%) were detected as heterozygous and no additional SNP was found close to target SNP by sequencing, while these sites were identified as homozygous after SNPlex analysis; iv) Class IV: 3 (3.3%) were homozygous after SNPlex analysis, while sequencing detected them as heterozygous with the presence of a third allele in respect to the Pinot Noir genotype; v) Class V: 1 (1.1%) was homozygous after SNPlex analysis, while sequencing detected it as homozygous in respect to the Pinot Noir genotype; vi) Class VI: 15 (16.3%) were homozygous after SNPlex analysis, while sequencing detected them as homozygous for the other allele variant present in the Pinot Noir genotype.

## Discussion

This study was intended to validate the use of 137 SNP markers, developed from the heterozygous genome of Pinot Noir clone ENTAV 115. The transferability of these markers was assessed across *V. vinifera *cultivars, wild forms of *V. vinifera*, and non-*vinifera Vitis *species to validate their utility as informative tools for marker-assisted selection in grape improvement programs, diversity studies, association analysis and mapping purposes.

### SNP transferability and informativeness

SNPs are more abundant in the genome and are more stably inherited than other genetic markers [35]. SNP detection does not involve gel electrophoresis, which is relatively slow and labour intensive, thereby it is more suitable for high throughput genotyping methods [36]. In addition to the SNPlex™ Genotyping System (Applied Biosystems Inc., [32]) used in this study, a wide array of technologies have now been developed, including chip-based (Affymetrix, [37]) and BeadArray (Illumina Inc., [38]) technologies, all providing efficient and reliable methods for large scale SNP surveys.

SNPs can be used for a range of purposes, including rapid identification of cultivars, construction of ultra high-density genetic maps, and association studies between a given genotype and a trait of interest [39]. In this study SNP transferability in the strict sense was tested because each of the analysed *Vitis *species is highly heterozygous and also because for several purposes (e.g. mapping) only heterozygous markers can be used. In this work it was found that 31.5% of the polymorphisms derived from Pinot Noir are maintained among unrelated *V. vinifera *cultivars, which is consistent with a survey across the cultivars Pinot Noir, Syrah, Grenache, Cabernet Sauvignon, and Riesling [23]. The heterozygous state transferability of 52.9% between Pinot Noir and genotypes related to Pinot Noir at the 1^st ^degree level was expected, without prior knowledge of the other parental genotypic state. Between Pinot Noir and genotypes related to Pinot Noir at the 2^nd ^degree level, a SNP transferability of 41.3% was obtained according to the possibilities derived from three unknown genotypic states in the phylogenetic tree. These results confirm the utility and the robustness of SNP analysis when dealing with *V. vinifera *cultivars, particularly in consideration of the cost and labour required for a resequencing strategy. However, the transferability of these SNPs to wild forms of *V. vinifera *was lower (18%), which will impact their use for association studies or selective sweep identification, and the transferability was even lower (2.3%) in non-*vinifera Vitis *species, which will affect their use for comparative mapping (Table 1). This latter result suggests that when working with non-*vinifera Vitis *species it will be important to employ a resequencing strategy while noting that the homology in priming sites was quite good. The fact that the PCR primers (for sequencing) developed based on a *V. vinifera *cultivar (Pinot Noir) matched the sequence of most *Vitis *species provides a valuable tool to get SNP and haplotype information useful for future diversity and association studies.

Marker informativeness can be evaluated using a number of different criteria. The number of alleles is the most basic criterion, where markers with a larger number of alleles are more likely to be polymorphic for any given germplasm set. Minor allele frequency (MAF) is a measure used to assess informativeness of SNP loci and is related to expected heterozygosity where the number of alleles is two, as is usually the case for SNPs [40] and was confirmed in this study. In fact, the occurrence of a triallelic SNP was very rare and was in accord to a previous SNP based study in grape [23].

SNPs with MAF values ≥ 0.05 or 0.10 are considered common and useful for most applications [e.g. [35,41,42]], whereas SNPs with MAF values ≥ 0.30 are the most informative and transferable across various genotypes [27]. In this study, the three highly informative (MAF ≥ 0.30) subsets of transferable markers developed for *V. vinifera *cultivars (72 SNPs), for wild forms of *V. vinifera *(50 SNPs), and for non-*vinifera Vitis *species (4 SNPs) represent new and valuable genomic tools for tasks such as marker-assisted breeding and genetic mapping. For genetic mapping one important criterion is that markers are transferable among pedigrees and ideally among species [43]. In the present study, of 137 SNPs only 1 marker was fully shared among all *Vitis *subgroups, in addition to pair wise shared SNPs. There were indeed 50 SNPs specific to *V. vinifera *cultivars, 17 specific to wild forms of *V. vinifera*, and 1 specific to non-*vinifera Vitis *species, which should be useful for accession characterization (fingerprinting) [see Additional file 3].

Additional transferability tests of these 137 SNP markers, developed based on the highly heterozygous Pinot Noir clone ENTAV 115 [6,30], were carried out *in silico *against the near-homozygous PN40024 genome sequence [5]. As expected, all the SNP regions were homozygous in PN40024 and the location of most of these was conserved between the two genomes [see Additional files 4 and 5].

### SNPlex validation through sequencing

The SNPlex genotyping system is a comprehensive solution for medium to large-scale genotyping studies, such as fine scale mapping, association analysis and marker-assisted breeding. Based on oligonucleotide ligation/polymerase chain reaction, it uses capillary electrophoresis to separate selectively amplified gene regions, reports easy-to-survey cluster plots, and manages large numbers of datasets [32]. Although the efficiency of 61% was good in this study, 39% of the assays failed. This percentage of failed assays is dependent on different assay-specific factors, such as DNA quality and DNA/probe interaction, and is slightly higher than in other SNPlex genotyping studies ([30]; Fischer *et al*. and Ward *et al*., 3^rd ^SNPlex user meeting 2008). One possible factor could be the multiple taxonomic levels compared in this study (cultivar, subspecies, species, genus) while other studies have used more simplified study sets.

Using the same approach as reported by Pindo *et al*. [30], SNPlex data were checked in 35 regions by sequencing. All data were confirmed, except for a few (3.8%) discrepancies. Most of these discrepancies (2.1% -heterozygous only in SNPlex; class I) reflected a preferential annealing of PCR primers caused by polymorphism (SNP or In/del) in the PCR priming site, which was identified by sequencing. Preferential amplification of one allele is a phenomenon widely described in the literature and it is expected to increase with the use of genetically diverse study sets [e.g. [44]], and most of the cases were detected in assays of *Vitis *species. The remaining cases (1.7%) were of the greatest interest, because they represented inconsistencies due to SNPlex failure, and limitations of this genotyping system. Possible explanations of the observed discrepancies may be: a preferential ligation of one SNPlex probe, given an additional SNP close to the target SNP (class II; also reported in Pindo *et al*. [30]); a ligation of only one SNPlex probe since the actual second allele is different from that of the reference Pinot Noir genotype (class IV); a non-specific SNPlex probe ligation (classes V and VI), even though a sequencing mistake could not be excluded (as well as for class III). To support these hypotheses, a long-range sequencing of three SNP regions was performed in all 69 *Vitis *genotypes. The results validated the class I hypothesis, given the identification of SNPs and In/dels in the short-range sequencing primer region, and confirmed all the previous short-range sequencing results.

Finally, no significant positive relationship was found when correlating SNPlex/sequencing inconsistencies (SNPlex failure classes II, III, IV, V and VI) to the average SNP and In/del frequency in each resequenced region (data not shown) and the reliability of the SNPlex assay in a genetically diversified sample was confirmed.

### Frequency of the SNPs

The SNP frequency detected by the short-range sequencing of the 35 regions found that cultivars of *V. vinifera *have one SNP for every 117 bp in coding and every 91 bp in non-coding regions. The results in coding regions match reports for other *V. vinifera *cultivars (1/115 bp, [25]; 1/127 bp, [26]). Other studies have found lower frequencies in non-coding regions (1/39 bp, [26]), but their samples were chosen to represent greater diversity. The frequency of one SNP every 169 bp of coding and every 89 bp in non-coding regions in the wild forms of *V. vinifera *were not comparable to a previous study of 9 *V. vinifera *cultivars and 2 wild forms of *V. vinifera *[27], emphasizing the dependency of these types of data on the chosen set of germplasm. The non-*vinifera Vitis *species had frequencies of one SNP every 112 bp in coding and every 82 bp in non-coding regions. These coding region results are in accord with values reported for *V. riparia *(1/73 bp, [25]), but no comparison exist for non-coding regions innon-*vinifera Vitis *species. SNPs were more prevalent in non-coding regions than in coding regions in all of the *Vitis *groups. This is in accord with reports for the heterozygous Pinot Noir sequence where coding and non-coding regions demonstrated different degrees of polymorphism (one SNP every 250 bp and every 182 bp, respectively, [6]).

In most organisms studied to date, SNPs are more prevalent in the non-coding regions of the genome, in fact the frequency of SNP distribution has been shown to vary not only among species, but also within each genome [38]. The SNPs detected in this study were primarily due to base transitions (64.7%, average among the three groups), which is consistent with previous results in grape [23,25] and in other organisms [45]. In addition to SNPs, In/dels were also detected. The heterozygous In/dels were more frequent than the homozygous In/dels in each of the three *Vitis *groups. No substantial difference in In/del occurrence was detected between coding and non-coding regions of each group, although this result is highly influenced by the chosen regions. There has not yet been a comprehensive study of In/del distribution in *Vitis *to which the results of this study can be compared. The Pinot Noir heterozygous genome has an average frequency of one In/del every 450 bp [6], which corresponds to the results reported for the three *Vitis *groups.

## Conclusion

In conclusion, using this SNPlex application and corroboration from the sequencing analysis, the informativeness (MAF information) of SNP markers from the heterozygous grape cultivar Pinot Noir is validated in *V. vinifera*, has a more limited application for wild forms of this species, and has no direct application for non-*vinifera Vitis *species The SNPlex technology was again validated as a robust method for rapid analysis of a limited number of SNPs on a large number of plants. Although additional SNPs could be used, the SNPs developed in this study will be very useful for accession identification and genomic-research assisted breeding at the *V. vinifera *level.

## Authors' contributions

SV carried out the genomic DNA extraction and the WGA, performed the sequencing data analysis, contributed to the SNPlex data analysis and drafted the manuscript. DM provided support in the SNPlex assay, carried out the sequencing data analysis and contributed to the manuscript writing. SR carried out the genomic DNA extraction and the SNPlex assay, contributed to the WGA and the SNPlex data analysis and helped in the discussion of the results. MP provided support in the SNPlex assay, performed the SNPlex data analysis and helped in the sequencing data analysis. RV contributed to reviewing the manuscript. PT provided important plant material and critically contributed to the discussion of the results. MAW provided basic plant material, helped in the introduction draft and in the discussion of the results, and contributed to reviewing the manuscript. MT performed the sequencing data analysis and critically contributed to the discussion of the results. RV conceptualized the project and contributed to the discussion of the results.

All authors read and approved the final manuscript.

## Supplementary Material

Additional file 1 **Table S1**. List of the studied 71 grape accessions. Click here for file

Additional file 2 **Table S2**. Linkage group, gene location and primer sequence of the 35 resequenced SNP regions. Click here for file

Additional file 3 **Table S3**. Linkage group, SNPlex results and allele frequencies of the 137 successful SNPs in all 71 grape accessions. Click here for file

Additional file 4 **Table S4**. BLAST-N results of the 137 SNP-regions – the 25 bp targeted by SNPlex – of Pinot Noir (clone ENTAV 115) on the PN40024 grape genome. Click here for file

Additional file 5 **Supplementary Text**. Details of the BLAST-N analysis of the 137 SNP regions on the PN40024 genome sequence. Click here for file
