# Supplementary material for: A SNP transferability survey within the genus Vitis
Source: BMC Plant Biol. 2008 Dec 16;8:128. doi: 10.1186/1471-2229-8-128 (PMC2631481; doi:10.1186/1471-2229-8-128)
Supplement: Additional file 5 — Supplementary Text. Details of the BLAST-N analysis of the 137 SNP regions on the PN40024 genome sequence. [file 1471-2229-8-128-S5.doc]

**Supplementary Text**

Given the availability of a near-homozygous grapevine (PN40024) genome [5], an additional transferability test of the 137 SNPs identified first in Pinot Noir clone ENTAV 115 [6, 30] was carried out *in silico*. The PN40024 is a genotype derived from Pinot Noir at the INRA station of Colmar by 9 successive selfing steps, with an outcross event likely by Helfensteiner (a cross between Pinot Noir and Frankenthal).

The BLAST-N analysis of the 137 SNP regions (only the 25 bp targeted by SNPlex) on the PN40024 genome sequence found a match for all SNP regions, except for four (SNP6110, SNP7075, SNP7092, SNP8115); these could correspond to regions either not assembled or absent in PN40024 [see Additional file 4]. The 133 heterozygous sites (SNPs) with hit were found to be homozygous in PN40024, in accord to its high degree of homozygosity. Of these 133, two SNP regions (SNP7268 and SNP7290) targeting one contig each (VV78X234154.16 and VV78X081674.11, respectively) in the Pinot Noir clone ENTAV 115 presented a double hit in the PN40024 genome [see Additional file 4]. This could be due to either misassembly or difference between the two genomes.

When comparing the positions of the SNPs in the two genomes (Pinot Noir clone ENTAV 115 and PN40024), most (99) of the SNPs with hit (133) did show a consistent localization. Moreover, very few (5, green) position inconsistencies were detected and they could be due to the differences between the two genome assembly strategies. Finally, in several cases (29, yellow) the precise location was available in either one or the other genome [see Additional file 4].
